# Relationship Between Macular Thickness and Visual Acuity in the Treatment of Diabetic Macular Edema With Anti-VEGF Therapy: Systematic Review

**DOI:** 10.1177/24741264221138722

**Published:** 2022-11-18

**Authors:** Patrick Wang, Zoe Hu, Maggie Hou, Patrick A. Norman, Eric K. Chin, David RP Almeida

**Affiliations:** 1 Department of Ophthalmology, Queen’s University, Kingston, ON, Canada; 2 Department of Radiology, Queen’s University, Kingston, ON, Canada; 3 Faculty of Medicine, University of British Columbia, Vancouver, BC, Canada; 4 Kingston General Health Research Institute, Kingston, ON, Canada; 5 Retina Consultants of Southern California, Redlands, CA, USA; 6 Loma Linda University Medical Center, Veterans Affair Hospital, Loma Linda, CA, USA; 7 Erie Retinal Surgery, Erie, PA, USA

**Keywords:** visual acuity, diabetic macular edema, central macular thickness, optical coherence tomography

## Abstract

**Purpose:**

To examine the relationship between central macular thickness (CMT) measured by optical coherence tomography (OCT) and visual acuity (VA) in patients with center-involving diabetic macular edema (DME) receiving antivascular endothelial growth factor (anti-VEGF) treatment.

**Methods:**

Peer-reviewed articles from 2016 to 2020 reporting intravitreal injections of bevacizumab, ranibizumab, or aflibercept that provided data on pretreatment (baseline) and final retinal thickness (CMT) and visual acuity (VA) were identified. The relationship between relative changes was assessed via a linear random-effects regression model controlling for treatment group.

**Results:**

No significant association between the logarithm of the minimum angle of resolution (logMAR) VA and CMT was found in 41 eligible studies evaluating 2667 eyes. The observed effect estimate was a 0.12 increase (95% CI, −0.124 to 2.47) in logMAR VA per 100 µm reduction in CMT after treatment change. There were no significant differences in logMAR VA between the anti-VEGF treatment groups.

**Conclusions:**

There was no statistically significant relationship between the change in logMAR VA and change in CMT as well as no significant effect of the type of anti-VEGF treatment on the change in logMAR VA. Although OCT analysis, including measurements of CMT, will continue to be an integral part of the management of DME, further exploration is needed on additional anatomic factors that might contribute to visual outcomes.

## Introduction

Center-involving diabetic macular edema (DME) is a leading cause of vision loss among diabetic patients in developed countries.^1^ In the past 2 decades, intravitreal vascular endothelial growth factor (VEGF) inhibitors have emerged as a first-line treatment, with efficacy based on visual acuity (VA) measurements and structural improvement on optical coherence tomography (OCT).^2^ The monitoring of clinical and anatomic factors, such as OCT central macular thickness (CMT), in conjunction with VA is often used to guide decision-making in determining the treatment frequency for patients with DME.^3^

Although OCT macular thickness, specifically CMT, is commonly used as a surrogate marker for clinical outcome measures such as VA, studies of the correlation between the 2 have produced conflicting findings.^3–6^ In general, previous studies have found modest correlations between VA and retinal thickness.^6–8^ As such, a comprehensive understanding of OCT-measured parameters in DME and the clinical implications for VA is paramount. We present a systematic review examining the relationship between OCT-measured CMT and VA in patients with DME receiving anti-VEGF treatment.

## Methods

### Literature Sourcing and Search Strategy

MEDLINE (Ovid), Embase, and Cochrane Central Register of Controlled Trials (CENTRAL) databases were searched to identify peer-reviewed studies published from 2016 to 2020 of patients with DME receiving anti-VEGF treatment that reported both VA and CMT before and after treatment. Search strategies were created for each database, and the searches were run on July 19, 2020. Database-specific subject headings for “Optical Coherence Tomography,” “Diabetic Retinopathy,” “Macular Edema,” and “Visual Acuity” and keywords for “retinal thickness,” “macular thickness,” “foveal thickness,” “diabetic macular edema,” and “DME” were used in the search strategy. Searches were modified to accommodate the syntax of each database.

### Inclusion/Exclusion Criteria

This review included all studies that met the following criteria: (1) at least 25 participants with DME and no other ocular disease; (2) intravitreal anti-VEGF injection as the primary intervention; (3) reported sufficient data for a comparison of pretreatment and post-treatment VA and CMT of patients with DME. All included studies also had at least a 1-month follow-up assessment.

The VA was recorded in logarithm of the minimum angle of resolution (logMAR) format; if only letter scores were presented, these were converted to the equivalent logMAR score using the equation 1.7 − (0.02 × ETDRS [Early Treatment of Diabetic Retinopathy Study] letter score). Some studies included data for patients receiving retreatment injections; however, these were included only if the previous treatment was administered at least 3 months before the latest intervention (with at least a 1-month follow-up).

Studies that were not in English or that examined nonhuman subjects were excluded. Also excluded were studies of participants with coexisting ocular conditions, such as age-related macular degeneration, proliferative diabetic retinopathy (DR), or retinal vein occlusion. Clinical trials, randomized trials, observational studies, retrospective studies, and prospective studies were included.

### Screening and Data Extraction

Two authors (P.W., Z.H.) independently performed the screening process. First, the titles and abstracts from the search were screened to identify eligible studies. Afterward, full-text screening was performed, and studies meeting all eligibility criteria were included. Cohen’s kappa coefficient was computed to determine the level of agreement between the 2 reviewers at each stage of screening. Mutual discussion between the authors was used to resolve conflicts in screening.

Data extracted from the studies that remained after full-text screening included the number of eyes, study design, study location and year, mean age of patients, proportion of male to female patients, dose and frequency of injection, total number of injections, length of follow-up, logMAR VA before and after treatment, and CMT before and after treatment.

### Statistical Analysis

Statistical analysis was performed with SAS software (version 9.4, SAS Institute). Most changes in the best-corrected VA (BCVA) and CMT from before to after treatment were reported as the mean and SD. If the SD was not reported, the value was extrapolated from the reported CIs. An analysis of the CMT and BCVA was performed using a linear regression model allowing for a random study intercept. The model was used to assess the relationship of the change in VA and CMT from before treatment (baseline) to after treatment. A 95% CI that did not cross zero was used to indicate statistical significance. Linear regression modeling adjusted for the baseline treatment group was also performed. Additional subgroup analysis by other baseline factors was planned for hemoglobin A_1c_ (HbA_1c_) level and age; however, these analyses could not be completed because of the limited reporting of data stratified by these categories.

## Results

### Overall Characteristics of Studies

The initial search yielded 2863 studies. After duplicates were removed, 2259 studies remained. After title and abstract screening, 762 full-text studies were assessed for eligibility. After full-text screening, 41 articles were deemed eligible for planned analyses comparing CMT with VA and were included in the review.^9–49^ The most common reasons for exclusion were a lack of participants and an incomplete dataset (eg, reporting percentage changes or for a fraction of participants only). Table 1 shows an overview of the included studies.

**Table 1.** Overall Study Characteristics and Reported BCVA and CMT Before and After Treatment.

		Diabetes Distribution, n (%)		Sex, n (%)			Mean ± SD							
Author^a^ (Country)	N	Type 1	Type 2	Male	Female	Mean Age (Y)	Baseline BCVA (LogMAR)	Baseline CMT (μm)	Post-treatment BCVA (LogMAR)	Post-treatment CMT (μm)	Treatment	FU (Mo)	Anti-VEGF Dose (mg)	Mean Inject (n)
Akpolat et al^9^ (Turkey)	31	NR		12 (39)	19 (61)	59.9	0.91 ± 0.65	431.65 ± 108.19	0.68 ± 0.53	371.66 ± 140.87	IVB	6	1.25	3
Al-Laftah et al^10^ (Qatar)	45	1 (2)	44 (98)	22 (58)	16 (42)	57.5	0.64 ± 0.36	444.95 ± 127.36	0.6 ± 0.32	272.85 ± 36.18	IVB	3	1.25	1
Bek and Jørgensen^11^ (Denmark)	64	6 (14)	38 (86)	26 (59)	18 (41)	64.2	0.45 ± 0.29	495.7 ± 125	0.38 ± 0.288	322.51 ± 73.96	IVR	7	0.30	3
Bezzina and Carbonaro^12^ (Malta)	65	0	65 (100)	41 (63)	24 (37)	64.26	0.6 ± 0.28	443.22 ± 121.04	0.53 ± 0.24	279.3 ± 50.6	IVB	3	1.25	3
Chen et al^13^ (Taiwan)	72	NR		55 (76)	17 (24)	58.6	0.61 ± 0.32	442.7 ± 84.3	0.54 ± 0.38	357 ± 147	IVA	3	2.00	3
Chung et al^14^ (Korea)	61	NR		36 (59)	25 (41)	54.9	0.9 ± 0.69	433 ± 146	0.43 ± 0.37	280.9 ± 12.6	IVB	1	1.25	1
Dursun et al^15^ (Turkey)	60	NR		26 (43)	34 (57)	61.86	0.54 ± 0.31	447 ± 147	0.39 ± 0.3	394 ± 148	IVR	3	1.00	3
El Shafei et al^16^ (Qatar)	45	NR		22 (58)	16 (42)	59.7	0.25 ± 0.14	444.95 ± 127.36	0.21 ± 0.11	389 ± 140	IVB	3	1.25	1
Faghihi et al^17^ (Iran)	42	20 (48)	22 (52)	20 (48)	22 (52)	59	0.7 ± 0.31	356 ± 116	0.7 ± 0.31	374.4 ± 73.5	IVB	4	1.25	1
Fang et al^18^ (Japan)	29	NR		18 (64)	10 (36)	61.7	0.76 ± 0.33	632.4 ± 196	0.6 ± 0.33	303.4 ± 32.16	IVB	3	1.25	1
Filek et al^19^ (Canada)	30	NR		17 (57)	13 (43)	63.9	0.53 ± 0.35	341.5 ± 55.33	0.33 ± 0.15	352 ± 52.8	IVR	24	0.50	3
Fouda and Bahgat^20^ (Egypt) Cohort 1	35	NR		NR		55.05	0.77 ± 0.23	465.29 ± 33.7	0.38 ± 0.25	359.3 ± 132.6	IVA	12	2.00	2.62
Fouda and Bahgat^20^ (Egypt) Cohort 2	35	NR		NR		56.64	0.74 ± 0.16	471.5 ± 34.4	0.43 ± 0.27	312.67 ± 115.38	IVR	12	0.50	2.62
Fu et al^21^ (China)	27	NR		NR		NR	0.8 ± 0.27	373.25 ± 59.39	0.38 ± 0.13	275.83 ± 82.38	IVR	6	0.50	3
Ghanbari et al^22^ (Iran)	42	NR		24 (57)	18 (43)	62.98	0.81 ± 0.38	553.88 ± 173.55	0.723 ± 0.38	338.1 ± 113.8	IVB	1	1.25	1
Hanhart et al^23^ (Israel)	35	16 (46)	19 (54)	24 (69)	11 (31)	63.8	0.49 ± 0.37	480 ± 175	0.42 ± 0.3	357 ± 145	IVB	8	1.25	1
Haritoglou et al^24^ (Germany)	51	NR		25 (49)	26 (51)	64.1	0.86 ± 0.38	501 ± 163	0.84 ± 0.41	302 ± 115	IVB	3	1.25	1
Hu et al^25^ (China)	113	NR		58 (51)	55 (49)	58.2	0.64 ± 0.23	409.5 ± 173.1	0.43 ± 0.17	309.8 ± 168	IVR	6	0.50	3
Tareen et al^26^ (Iran)	78	NR		25 (60)	17 (40)	53.5	0.42 ± 0.14	452.9 ± 143.1	0.16 ± 0.14	421.4 ± 192.76	IVB	6	1.25	3
Kaiho et al^27^ (Japan)	51	NR		27 (53)	24 (47)	64.7	0.39 ± 0.3	489.6 ± 106.8	0.3 ± 0.28	471.45 ± 129.69	IVA	12	1.25	12
Kim et al^28^ (Korea) Cohort 1	29	NR		16 (55)	13 (45)	60.25	0.54 ± 0.36	377.1 ± 145.9	0.49 ± 0.34	349 ± 85	IVB	12	1.25	3
Kim et al^28^ (Korea) Cohort 2	21	NR		13 (62)	8 (38)	57.42	0.23 ± 0.16	427.7 ± 143.1	0.15 ± 0.11	336.1 ± 166.6	IVB	12	1.25	3
Kim et al^28^ (Korea) Cohort 3	15	NR		6 (40)	9 (60)	59.37	0.19 ± 0.08	485.1 ± 187.1	0.17 ± 0.12	421.4 ± 192.76	IVB	12	1.25	3
Konidaris et al^29^ (UK)	49	NR		34 (69)	15 (31)	67.48	0.71 ± 0.3	432.58 ± 163.72	0.58 ± 0.18	390.86 ± 125.92	IVA	6	1.25	6
Kook et al^30^ (Germany)	126	NR		65 (52)	61 (48)	66.1	0.82 ± 0.37	463 ± 173	0.74 ± 0.34	447 ± 174	IVB	12	1.25	1
Kook et al^31^ (Germay)	30	NR		17 (57)	13 (43)	67	0.72 ± 0.36	332 ± 136	0.59 ± 0.31	326.51 ± 175.06	IVB	9	1.25	1
Koytak et al^32^ (Turkey) Cohort 1	42	NR		15 (36)	27 (64)	57.21	0.75 ± 0.48	366.98 ± 121.45	0.61 ± 0.46	374.4 ± 73.5	IVB	1	1.25	1
Koytak et al^32^ (Turkey) Cohort 2	31	NR		11 (35)	20 (65)	59.29	0.67 ± 0.47	463.32 ± 120.69	0.53 ± 0.44	309.8 ± 168	IVB	1	1.25	1
Koytak et al^32^ (Turkey) Cohort 3	19	NR		8 (42)	11 (58)	58.95	0.88 ± 0.57	515.05 ± 165.83	0.79 ± 0.6	302 ± 115	IVB	1	1.25	1
Lai et al^33^ (Taiwan)	119	NR		43 (48)	46 (52)	61.82	0.74 ± 0.3	446.5 ± 117.6	0.64 ± 0.37	268 ± 74	IVR	12	0.50	3
Laiginhas et al^34^ (Portugal)	49	4 (8)	44 (92)	31 (63)	18 (37)	65.8	0.55 ± 0.32	473 ± 146	0.46 ± 0.33	350 ± 152	IVA	2.4	2.00	2.2
Lam et al^35^ (Hong Kong) Cohort 1	23	NR		11 (42)	15 (58)	65.4	0.63 ± 0.31	453 ± 128	0.52 ± 0.31	324.5 ± 116.4	IVB	6	1.25	3
Lam et al^35^ (Hong Kong) Cohort 2	25	NR		17 (65)	9 (35)	65.6	0.6 ± 0.28	468 ± 113	0.47 ± 0.26	405.9 ± 128.4	IVB	6	2.50	3
Laugesen et al^36^ (Denmark)	33	10 (30)	23 (70)	18 (55)	15 (45)	63	0.57 ± 0.29	412 ± 64.5	0.54 ± 1.2	366.82 ± 105.13	IVR	3	0.50	1
Lee et al^37^ (Korea)	90	NR		59 (69)	26 (31)	61.2	0.54 ± 0.33	468.1 ± 105	0.49 ± 0.31	283 ± 94	IVB	3	2.50	1
Lee et al^38^ (Korea)	60	NR		32 (53)	28 (47)	53.1	0.53 ± 0.51	431 ± 412.1	0.44 ± 0.43	324.6 ± 70.2	IVB	2	1.25	1
Lee et al^39^ (Korea)_2	31	NR		18 (58)	13 (42)	57.3	0.33 ± 0.28	396.8 ± 111.3	0.21 ± 0.18	360.8 ± 85.7	IVB	3	1.25	3
Lim et al^40^ (Korea)	38	NR		19 (50)	19 (50)	61.4	0.62 ± 0.23	447 ± 110	0.46 ± 0.28	346.22 ± 108.24	IVB	12	1.25	2
Nepomuceno et al^41^ (Brazil) Cohort 1	32	NR		13 (41)	19 (59)	63.8	0.6 ± 0.05	451.7 ± 22.3	0.36 ± 0.05	296.6 ± 80.4	IVB	11	1.50	9.84
Nepomuceno et al^41^ (Brazil) Cohort 2	28	NR		14 (50)	14 (50)	63.7	0.63 ± 0.06	421.9 ± 23.1	0.34 ± 0.04	291 ± 41.3	IVR	11	0.50	7.67
Ozcaliskan et al^42^ (Turkey) Cohort 1	37	NR		23 (62)	14 (38)	64.1	0.46 ± 0.35	403.46 ± 77.77	0.35 ± 0.31	352.06 ± 109.62	IVA	12	2.00	3
Ozcaliskan et al^42^ (Turkey) Cohort 2	38	NR		25 (66)	13 (34)	63.31	0.59 ± 0.34	516.97 ± 149.18	0.47 ± 0.34	395.26 ± 163.17	IVA	12	2.00	3
Ozcaliskan et al^42^ (Turkey) Cohort 3	40	NR		22 (55)	18 (45)	64.92	0.47 ± 0.21	448.5 ± 110.05	0.4 ± 0.31	389 ± 140	IVA	12	2.00	3
Rahimy et al^43^ (USA)	50	NR		19 (51)	18 (49)	69.6	0.6 ± 0.43	459.2 ± 139.2	0.55 ± 0.48	349 ± 85	IVA	2.5	2.00	2
Schiefelbein et al^44^ (Germany)	112	NR		68 (61)	44 (39)	62.77	0.533 ± 0.369	469 ± 171	0.47 ± 0.363	329.7 ± 19.3	IVR	12	1.25	3
Seo and Park^45^ (Korea)	30	5 (17)	25 (83)	18 (60)	12 (40)	57.9	0.73 ± 0.36	498.96 ± 123.99	0.61 ± 0.4	401.2 ± 155.4	IVB	1	1.25	1
Seo et al^46^ (Korea) Cohort 1	23	NR		12 (52)	11 (48)	60.05	0.4 ± 0.16	344.2 ± 50.83	0.28 ± 0.1	303.4 ± 32.16	IVR	12	1.25	3.69
Seo et al^46^ (Korea) Cohort 2	16	NR		6 (38)	10 (63)	54.91	0.6 ± 0.24	410.91 ± 135.66	0.34 ± 0.12	471.45 ± 129.69	IVR	12	1.25	5.33
Seo et al^46^ (Korea) Cohort 3	16	NR		6 (38)	10 (63)	56.92	0.55 ± 0.22	417.42 ± 136.19	0.48 ± 0.26	272.85 ± 36.18	IVR	12	1.25	5.09
Soheilian et al^47^ (Iran)	37	NR		17 (46)	20 (54)	60.9	0.78 ± 0.28	352 ± 140	0.53 ± 0.33	336.1 ± 166.6	IVB	3	1.25	1
Vyas et al^48^ (Nepal)	52	0	33 (100)	34 (65)	18 (35)	58.59	0.8 ± 0.46	449.03 ± 177.92	0.6 ± 0.42	387.3 ± 87.8	IVB	6	1.25	1
Yuksel et al^49^ (Turkey)	71	NR		36 (61)	23 (39)	59.2	0.88 ± 0.4	515.4 ± 150.3	0.8 ± 0.4	370 ± 147	IVB	6	1.25	1

Abbreviations: anti-VEGF, antivascular endothelial growth factor; BCVA, best-corrected visual acuity; CME, central macular thickness; FU, follow-up; Inject, injections; IVA, aflibercept; IVB, bevacizumab; IVR, ranibizumab; logMAR, logarithm of the minimum angle of resolution; NR, not reported.

a First author.

Overall, the total number of eyes included was 2667, with 1570 eyes in the intravitreal bevacizumab (IVB) group, 676 eyes in the intravitreal ranibizumab (IVR) group, and 421 eyes in the intravitreal aflibercept (IVA) group. All studies met the criteria of having follow-up of at least at 1 month, with the mean follow-up in all groups being 8.2 months—5.6 months, 10.1 months, and 12.8 months in the IVA, IVB, and IVR groups, respectively. Across all studies, the mean baseline BCVA was 0.62 ± 0.17 logMAR and the mean baseline CMT was 444 ± 55 µm, indicating considerable heterogeneity among the analyzed cohorts.

### Relationship Between Visual Acuity and Macular Thickness

**Figure 1.**
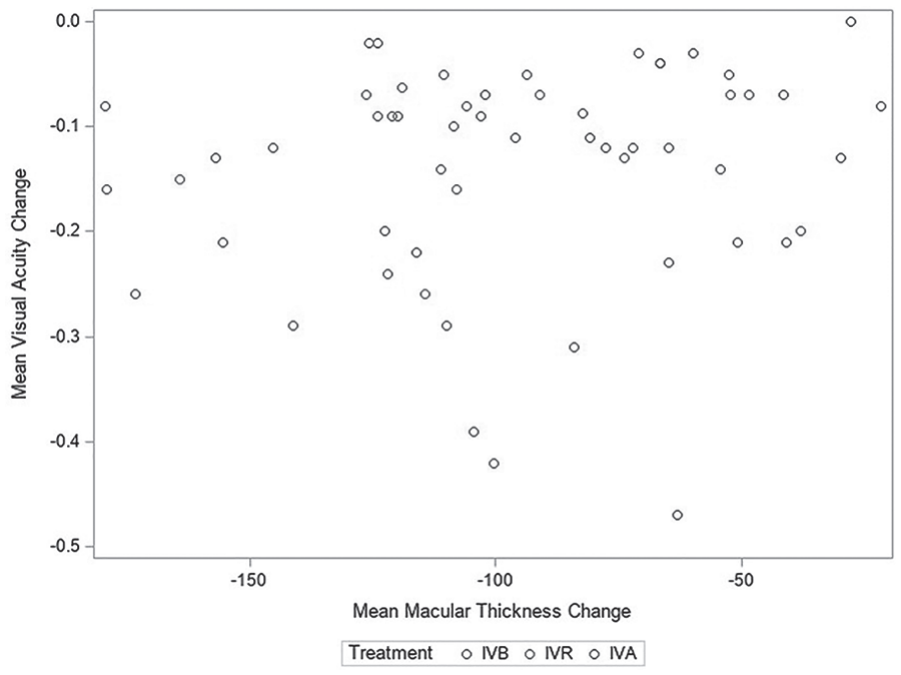
Scatterplot of absolute changes in best-corrected visual acuity and central macular thickness in all included studies. Abbreviations: IVA, intravitreal aflibercept; IVB, intravitreal bevacizumab; IVR, intravitreal ranibizumab.

**Figure 2.**
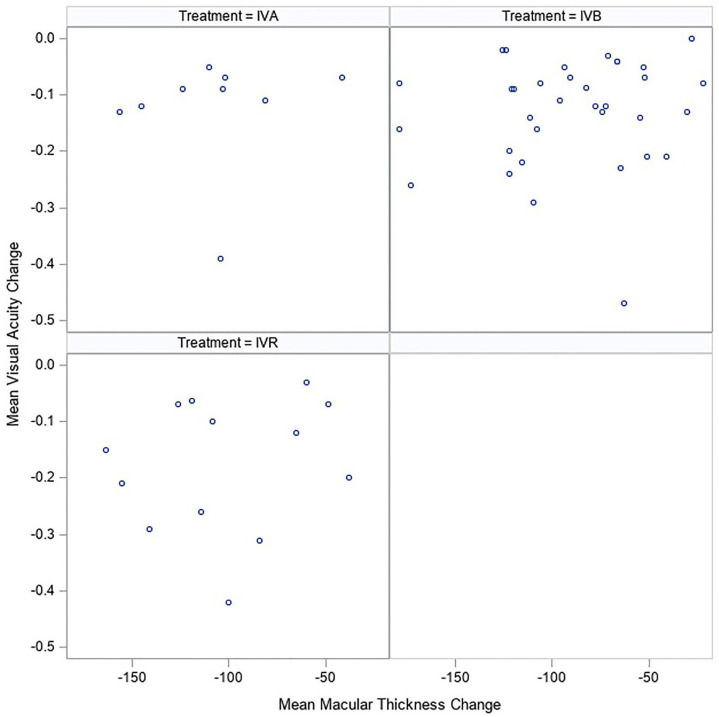
Scatterplot of absolute changes in best-corrected visual acuity and central macular thickness stratified by treatment. Abbreviations: IVA, intravitreal aflibercept; IVB, intravitreal bevacizumab; IVR, intravitreal ranibizumab.

Figure 1 and Figure 2 show the absolute changes in VA and CMT, respectively, in each study stratified by treatment type. Each figure shows the reported CMT change (µm) in relation to the change in BCVA. Overall, no significant association between logMAR VA and CMT was found, with an observed effect estimate of a 0.12 increase (95% CI, −0.124 to 2.47) in logMAR VA (7.9 ETDRS letters) after treatment per a 100 µm reduction in CMT change after treatment.

### Stratified Subgroup Analysis of Baseline Factors

Notable outliers were present in all treatment groups, and subgroup analysis was performed to identify additional correlations. When stratified by treatment subgroup (Figure 2), there was no significant difference between the 3 anti-VEGF treatment groups, with a difference of −0.017 logMAR VA (95% CI, −1.48 to 1.44) between the IVA group and IVR group and of 0.41 logMAR VA (95% CI, −0.56 to 1.39) between the IVB group and IVR group.

Three studies reported findings exclusively for eyes that did not receive previous medical treatment before the study period (treatment naïve).^14,20,22^ These studies were outliers with a higher mean baseline VA (0.82 ± 0.47 logMAR) than in the entire cohort (0.62 ± 0.17 logMAR) and a larger effect estimate of a 0.21 increase (95% CI, 0.16 to 0.26) in logMAR VA per 100 µm reduction in CMT.

## Conclusions

The aim of this systemic review was to examine the relationship between visual outcomes of VEGF antagonists for DME. We focused specifically on how the anatomic response (change in CMT) was correlated with the resultant change in VA. In our analyses of 53 studies, we found no statistically significant relationship between the change in logMAR VA and the change in CMT. There was also no significant effect of the type of anti-VEGF treatment on the change in logMAR VA. Although the change found in this study was not statistically significant, previous studies found a modest but significant correlation between VA and CMT.^6–8,50^ This ambiguity might make it difficult for clinicians to extrapolate exactly how many letters (or lines) of vision improvement might be expected without further research on the topic. We regard this as useful prognostic information that patients might find helpful in early interventions.

Previous studies of patients with DME treated with anti-VEGF agents showed a modest but significant correlation between VA and CMT, with a trend toward predicted direction and magnitude.^6–8,50^ Our review confirms a modest relationship between the 2 key metrics, with a lack of statistical significance between the reduction in CMT and improvement in VA.

Variable VA changes after intervention have also been reported. For instance, the 5-year results in a cross-sectional longitudinal study (Diabetic Retinopathy Clinical Research Network [DRCRN] Protocol I)^51^ showed that patients who received prompt laser and deferred ranibizumab had decreased macular thickness; however, their VA was worse. In addition, 2-year results in a randomized clinical trial of patients treated with bevacizumab and who had a VA of 20/40 (DRCRN Protocol T),^3^ the VA improved the same amount as with the other 2 anti-VEGF drugs, despite the increased CMT compared with that in the other 2 groups. Clinically, this translates to the takeaway that anatomic improvement does not always promise functional physiologic vision improvement. These results corroborate conclusions from one of the first major studies by the DRCRN in 2007, accepting that OCT measurements on their own are inadequate surrogates for VA measurements.^52^

In a study of DRCR Retina Network Protocol T,^4^ subgroup analysis was performed for baseline factors associated with VA change, including treatment group, treatment interaction, HbA_1c_ level, age, previous panretinal photocoagulation treatment, and DR severity. There were no statistically significant interactions between treatment group and the correlation between VA and CMT changes, which is corroborated by our review. We could not evaluate the association between HbA_1c_ level and age in this review due to limited reporting of stratified data.

Several factors might have affected the degree of association calculated in our study. Overall, heterogeneity in study designs as well as variable follow-up duration could have induced heterogeneity in the study outcomes. The notable outliers in the plotted studies and subgroup analysis were predominantly in originally treatment naïve patients.^14,20^ It is also possible that patients receiving initial anti-VEGF treatment have a larger decrease in CMT; however, chronic residual macular edema might persist.^51^ Moreover, accounting for anatomic perturbations, such as inner retinal disorganization, the presence of hyperreflective foci (eg, lipid exudates or intraretinal hemorrhages), the integrity of the outer retina (eg, external limiting membrane and photoreceptors), retinal atrophy, and ischemia might contribute more meaningfully to VA.

When incorporating OCT CMT metrics in DME management, it is important to consider whether any reported changes in CMT, with or without predictable changes in VA, are sustained. Although previous reviews found a modest correlation overall, the long-term benefits of reducing CMT to improve VA remains to be established.^20,53,54^ Consideration of external elements contributing to VA (eg, cataract and other media opacity) can have an undetermined impact.

Although our review was not intended to ascertain differences in treatment outcomes between anti-VEGF interventions, it has been well documented previously that VEGF antagonists appear to have a consistent effect on improving both VA and CMT.^3^ Nonetheless, we found no statistically significant relationship between the change in logMAR VA and the change in CMT; thus, overemphasis on CMT as the only outcome measure relevant to VA should be avoided. Although OCT analysis, including measurements of CMT, will continue to be an integral part of DME management, further exploration on additional anatomic factors that might contribute to visual outcomes is needed.
